# Difficult Therapeutic Decisions in Gorham-Stout Disease–Case Report and Review of the Literature

**DOI:** 10.3390/ijerph191811692

**Published:** 2022-09-16

**Authors:** Katarzyna Wojciechowska-Durczynska, Arkadiusz Zygmunt, Marta Mikulak, Marta Ludwisiak, Andrzej Lewinski

**Affiliations:** 1 Department of Endocrinology and Metabolic Disease, Medical University of Lodz, 93-338 Lodz, Poland; 2 Department of Endocrinology and Metabolic Disease, Polish Mother’s Memorial Hospital–Research Institute, 93-338 Lodz, Poland

**Keywords:** osteolysis, lymphogenesis, zoledronic acid, sirolimus, therapy

## Abstract

Gorham-Stout disease (GSD) is a very rare, life-threatening condition characterized by the proliferation of lymphatic vessels and osteolysis. Unfortunately, no standard treatment has been determined for management of GSD. The available therapies are not equally effective and carry substantial side-effects. We report a 42-year-old female with GSD manifested in multifocal osteolysis and chronic chylothorax and ascites. The combined treatment with sirolimus and zoledronic acid due to its synergism of action was introduced. To our knowledge, this is the first Polish case report of adult patients with Gorham-Stout disease.

## 1. Introduction

Gorham-Stout disease (GSD) is a very rare condition characterized by an extensive and progressive osteolysis. So far, several hundred cases have been described in the literature worldwide [1,2]. Herein, we describe the first adult case of GSD in Poland.

The etiology of this disease remains mostly unknown. Osteolysis results from the generalized proliferation of lymphatic vessels and elevated localized osteoclastic activity [3]. Evidence suggests that the growth factors (including VEGF-A, -C and -D) cause uncontrolled proliferation of lymphatic vessels [4,5,6,7]. These growth factors (especially VEGF-C) activate phosphoinositide-3-kinase (PI3K), resulting in signaling of AKT and the mammalian target of rapamycin (mTor), which ends in promotion of lymphogenesis, angiogenesis and cell proliferation [8,9]. Mammalian target of rapamycin is an important kinase in the progression of the cell cycle and a key regulator of immune responses. Therefore, mTor was also identified as an attractive therapeutic target in numerous neoplasm and vascular malformations [10].

The increased vascularity of the affected bone results in a high rate of oxygen consumption and alters tissue acidity [11]. Mechanism of osteoclast activation is still unclear. It has been suggested that the main contributory factors are increased sensitivity of osteoclast progenitor cells to osteoclast-inducing factors and receptor activator of nuclear factor ĸB ligand (RANKL) [12,13]. Furthermore, elevated levels of IL-6 which activate osteoclasts, have been recorded in patients with active GSD [14]. No definite correlations between genetic mutations and clinical manifestation have been found [2].

The risk of GSD is not restricted to gender (1.6:1–male to female ratio), race or geographical distribution [15]; it can also be diagnosed at any age, but it is most often diagnosed in children and young adults. There are no known environmental factors that would be associated with GSD [2]. However, sometimes the disease can occur after a traumatic event [16].

It may involve any sites of the body although commonly affects craniofacial area, shoulder and pelvic girdle, ribs, and spine [17]. GDS can arise in a single bone or in multiple contiguous bones [18]. The clinical presentation of GSD is highly variable depending on the structures involved. The most common symptom is local pain, and the first symptom of the disease might be pathological fracture of the affected bone.

The diagnosis is based on radiological examinations (progressive osteolysis and destruction of the cortical bone) and/or bone biopsy (immunohistochemical markers of lymphatic endothelial cells in the myeloid and cortical bone areas–podoplanin/D2-40) [19]. Furthermore, Chung et al. reported four different radiographic phases in GSD progression: 1st phase: presence of radiolucent foci; 2nd phase: the foci converge, generating new radiolucent areas; 3rd phase: cortical disruption with the invasion of adjacent soft tissues; 4th phase: replacement of bone tissue by fibrous tissue [20].

The complications of the disease are very serious. One of them is respiratory failure (caused by chylothorax due to excessive growth of pulmonary lymphatic channels) in cases with the involvement of the thoracic spine or ribs, and neurological consequences with paralysis and cerebrospinal fluid leakage in cases with vertebral involvement, as well as in the case of involvement of the cervical spine, the base of the cranium or craniofacial bones [21].

The prognosis of GSD is variable and depends on multiple factors such as extent and location of the affected areas. The mild cases of the disease can be self-limiting for many years, while severe cases involving the craniofacial (cerebrospinal fluid leakage) and/or thoracic regions (chylothorax) can be fatal. The lung tissue involvement may herald a deteriorating prognosis [2]. This article describes a rare case of multifocal GSD in an adult female patient and the difficult therapeutic decisions concerning this case. The patient was informed that data concerning the case would be submitted for publication and gave informed consent. A review of the relevant literature has also been included.

## 2. Case Description

In March 2021, a 42-year-old female patient with diagnosed GSD visited the Outpatient Clinic of Osteoporosis Treatment in the Polish Mother’s Memorial Hospital-Research Institute in Lodz. The first symptoms of the disease were observed in 1990, when the patient underwent left-sided thoracotomy due to pleural effusion (chylothorax). Another episode of chylothorax occurred in 2007. At that time, the symptoms were edema of the lower extremities and an enlarged circumference of the abdomen. The exploratory laparotomy was also performed, which showed presence of numerous angiomas in the abdominal cavity. However, the first diagnosis of GSD was made one year later in 2008 after another episode of chylothorax and due to results of chest computed tomography (CT) scan, which revealed an area of bone destruction in the thoracic spine and the sternum. At that time, the patient began to complain of severe bone pain with varying degrees of intensity, requiring the use of painkillers. In the same year, due to the fourth episode of chylothorax, the mechanical pleurodesis and right-sided talc pleurodesis were performed, complicated by an abscess of the right pleural cavity. Concomitantly, the thoracic spine MRI scans confirmed the area of osteolysis in the vertebral bodies and in the sternum manubrium. Since 2010, the patient has also experienced a gradual decrease in body weight. In 2010–2021, the patient experienced several consecutive episodes of lymphorrhea into the pleural and peritoneal cavities. In addition, due to the presence of uterine myoma and hypermenorrhea, in 2019 the patient underwent a hysterectomy. In March 2021, following endoscopic examination, hemorrhagic gastroduodenitis was diagnosed. Family history of bone disease was negative. The patient’s father died of colorectal cancer at the age of 55. The mother died of complications from pneumonia at the age of 75. One brother died of a hemorrhagic stroke; two other brothers of the patient died of complications from type 2 diabetes. Two remaining brothers are alive, one of them has type 2 diabetes. The patient gave birth twice, once in 1999 and once in 2002. The patient used to work as a shop assistant, and she is currently receiving a disability benefits. She denies smoking or abusing alcohol.

The patient did not receive any causal treatment during 2007–2021. In July 2021, the treatment with sirolimus was introduced.

In December 2021, the patient was hospitalized in the Department of Endocrinology and Metabolic Diseases of the Polish Mother’s Memorial Hospital-Research Institute. On admission the patient reported persistent, severe pain in the pelvic area, sternum and ribs requiring constant intake of opioid analgesics. The physical examination revealed that patient was underweight (BMI 17.16 kg/m^2^), numerous postoperative scars (abdomen, chest), silencing of the vesicular sound over the lower pulmonary fields, and signs of ascites.

Additionally, the patient was diagnosed with anemia, hemorrhagic gastroduodenitis, varicose veins of the gastric antrum and cholelithiasis. The patient did not have a history of fractures in past. Postmenopausal or secondary osteoporosis or inflammatory bone disease or malignant neoplasm should be considered in the differential diagnosis.

Further laboratory investigations such as routine blood examination, hormonal and biochemistry were performed. Most of the laboratory tests were within normal limits, including calcium, phosphate, parathormone, estradiol, follicle stimulating and luteinizing hormones, vitamin D, alkaline phosphatase, B-CrossLaps and osteocalcin levels (Table 1).

The densitometric examination revealed progressive bone loss (Table 2).

**Figure 1:**
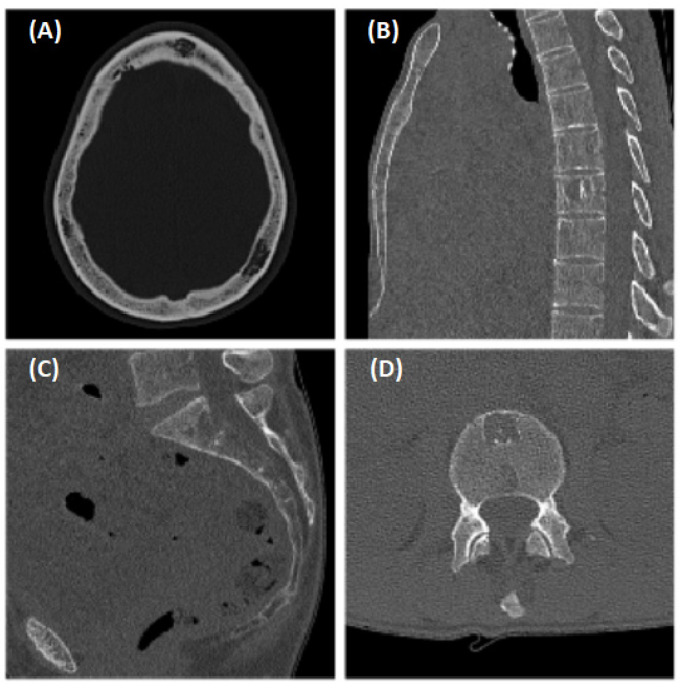
CT scans show areas of osteolysis: (**A**)—in the frontal and parietal bones of cranium; (**B**)—in thoracic vertebral body, Th8; (**C**)—in sacral vertebra body, S1; (**D**)—in lumbar vertebra body, L3.

**Figure 2:**
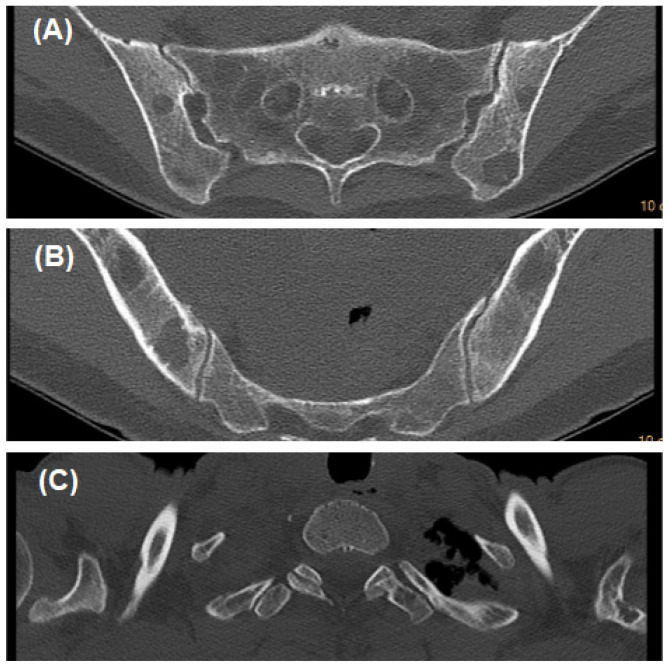
CT scans show areas of osteolysis: (**A**)—in pelvic girdle (the sacrum and iliac bones); (**B**)—in iliac bones; (**C**)—in left scapula.

The whole skeleton and SPECT/CT scintigraphy revealed area of osteolysis in the pelvis and the vertebrae. The CT scans of different parts of the body further revealed area of osteolysis in cranium, sacrum and iliac bones, thoracic, lumbar vertebrae and scapula (Figure 1 and Figure 2). Moreover, the chest CT scan revealed both sides pleural effusion fluid. In addition, the ultrasound examination of the abdominal cavity revealed the presence of fluid in the abdominal cavity and in minor pelvis.

The diagnosis of GSD was established after the exclusion of endocrine, inflammatory, and neoplastic disease and was based on the clinical manifestation and characteristic radiological image. There are no standardized recommendations for the diagnosis of GSD. Based on multidisciplinary guidelines for initial evaluation of complicated lymphatic anomalies, including GSD, the need for bone biopsy should be discussed after clinical and radiological evaluation, taking into consideration possible complications of biopsy [22]. In the case of our patient, the clinical manifestation was very characteristic for the disease and there was no clinical or laboratory suspicion for an alternative cause of osteolysis. Moreover, the patient did not consent to a bone biopsy.

In the patient reported by us, the disease is chronic and progressive. The first symptoms of the disease appeared 25 years ago in the form of lymphorrhea, which after 3 years was accompanied by bone pain indicative of osteolysis. Based on the available literature, it can be concluded that the evolution of the disease is very diverse, and the mechanism of evolution is related to proliferation of intramedullary and cortical vessels, and later stage characterized by the destruction and resorption of bone. Therefore, for the current case, we decided to apply combined treatment. Firstly, anti-angiogenic therapy with oral sirolimus at a dose of 2 mg per day was initiated in July 2021 to inhibit lymphatic vascularization. Secondly, because the patient was undergoing persistent progressive bone loss, the zoledronic acid at a dose of 4 mg iv was administered in December 2021, as a single intravenous injection. Written informed consent was obtained from the patient. Three months after drug administration, an improvement in bone density parameters was observed in the densitometric examination (Table 2). Further thorough clinical, laboratory and densitometric monitoring of the patient is planned.

## 3. Discussion

Since very little is known regarding GSD, no standard treatment has been determined for its management. Depending on the symptoms and disease progression, treatment attempts are made with the use of various modalities including surgery, radiotherapy, as well as more conservative treatment including medications that inhibit the proliferation of lymphatic vessels, such as sirolimus, interferon-alpha or interfere osteolysis, e.g., bisphosphonates, denosumab, calcitonin.

To date, the treatment of massive osteolysis is controversial. A variety of treatment modalities targeting control of progressive osteolytic activity have been assessed. A review of the literature allows for analysis of the problem. In the publication by Matsumoto et al. [23], a case of a 47-year-old man with massive osteolytic lesions and fractures in the left shoulder girdle was described. The bone biopsy revealed marked osteolysis and proliferation of atypical vessels. Decision to start treatment with intravenous pamidronate was made. After one year, the treatment was changed to orally administered alendronate for the next 4 years. At the follow-up, 9 years after the discontinuation of therapy, no clinical or radiological signs of disease progression were recorded [23]. The inhibition of local osteolysis using bisphosphonates is usually helpful to prevent local progressive osteolysis in GSD. However, in the case of our patient, the use of oral bisphosphonates was contraindicated due to hemorrhagic gastroduodenitis.

Another report by Illeez et al. [24] presents the case of a 55-year-old patient with a history of pain in the elbow, knee, and wrist joints for approximately 20 years. Due to the intensification of pain symptoms in the elbow joints, extended diagnostics was performed again and finally the diagnosis of Gorham’s disease was made. Treatment with zoledronic acid was introduced, resulting in a significant reduction in pain and improvement of bone density and structure in radiological examinations images.

Mo et al. [8] described the case of a 14-year-old boy with rapidly progressing scoliosis. Imaging examinations showed osteolysis around two ribs and spinous processes of the cervical and thoracic vertebrae. The treatment with sirolimus was administered preoperatively, then surgical treatment was performed. Postoperatively, sirolimus was reintroduced with the combination with zoledronic acid. The examinations performed after two years confirmed a partial reconstruction of the affected ribs and spine [8].

Another case of an 18-year-old patient with a very similar clinical course of GSD to our patient was reported by Cramer et al. [25]. The patient was diagnosed with osteolitic fractures in the X and IX ribs and a massive right-side pleural effusion. After confirming the diagnosis of GSD, the treatment with alendronate was introduced, but it proved ineffective. Treatment was modified to combined therapy with zoledronic acid and interferon alpha. This treatment halted progressive bone loss, however, the pleural effusion lingered. Treatment was re-modified once again and sirolimus was added to zoledronic acid obtaining satisfactory treatment results, i.e., withdrawal of pleural effusions and stopping of the progression of bone loss [25].

Treatment for this potentially fatal disease remains challenging and requires a multifaced approach to achieve stabilization. Sirolimus was discovered to have potent immunosuppressive and antiproliferative properties due to its ability to inhibit mTOR pathway. Zoledronic acid is a nitrogen-containing bisphosphonate that inhibits osteoclast-mediated bone resorption and induces apoptosis by inhibiting enzymes of the mevalonate pathway [26]. Zoledronic acid also inhibits activation of mTOR cascade, too [13]. Thus, a combination of therapy of zoledronic acid and sirolimus may lead to down regulation of mTOR pathway by two synergistic mechanisms.

Another case concerns a 27-year-old patient with long history of increasing painless mobility of the right upper incisors. The histopathological examination of the jawbone revealed bone resorption with the presence of proliferation of lymphatic vessels. GSD was diagnosed. The radiotherapy and pharmacotherapy with bisphosphonate WERE introduced, without any improvement. Therefore, pharmacotherapy with sirolimus and denosumab was administered. One month later, in the CT scans further progression of osteolysis with the involvement of the skull base on the right side, bone inflammation of the skull base, stroke of the brain stem with lateral bulb syndrome was found. Ultimately, the patient died after a few weeks [27]. Denosumab is a human monoclonal antibody to RANKL and its effectiveness in inhibiting bone resorption in case of GSD involving shoulder girdle has been presented [23]. However, data on the toxic combination of denosumab with anti-angiogenic drugs, such as sirolimus, can also be found in the literature. It has been observed that a side effect of such a combination is an increased risk of osteonecrosis of the jaw, therefore special care should be taken when combining these two drugs [28].

There are several treatment options and possible clinical scenarios relevant to our case. The progression of osteolysis is mostly unpredictable. In most patients, osteolysis progresses until entire bone is involved and adjacent soft tissues may also be invaded. However, in some patients, progression may be self-limiting and may experience a quiescent period during which bone resorption is stable and does not progress [11]. In current case, osteolysis progresses quickly, despite the fact that the patient is a premenopausal female and has no pathological fractures so far. The review of the available literature indicates that it is advisable to initiate treatment as soon as possible in order to avoid fractures and subsequent bone deformation and complications. It should also be noted that our patient’s clinical course with involvement of pleura (chylothorax) results in poor prognosis. Further complications, including nerve root compression can be expected. Chylothorax can also lead to progressive hypoproteinemia, malnutrition and immunosuppression with lymphocytopenia. In case of our patient, we have recorded cachexia (BMI < 18) from the beginning of observation. In addition, pulmonary or pleural involvement may lead to respiratory failure and death. Infection of relevant bones and septic shock is another rare complication of GSD that has been reported [21].

The case of GSD, presented by us, is the second report of the patient with this rare syndrome in the Polish population. The first report of other authors has described an 8-year-old boy with involvement of the left clavicle and scapula, however, there was no information on the applied therapy [29].

## 4. Conclusions

In conclusion, GSD is rare and well-known for not only its challenging diagnosis but also appropriate treatment. We have reported a case of rare GSD to alert physicians to clinical signs and symptoms and difficulties in the management of GSD. To prevent the resorption of affected bones and achieve complete healing we strongly recommend that early treatment should be introduced. Otherwise, further complications due to the disease development may drastically affect future prognosis of patients.

## Figures and Tables

**Table 1:** Laboratory tests during hospitalization.

	Parameters	Reference Range
Total calcium [mmol/L]	2.46	2.1–2.55
Ionized calcium [mmol/L]	1.30	1.2–1.32
Inorganic phosphates [mmol/L]	0.86	0.81–1.45
Magnesium [mmol/L]	0.73	0.7–1.0
Inorganic phosphates in 24-h urine collection [mmol/24 h]	6.60	12.9–42
Calcium in 24-h urine collection [mmol/24 h]	7.100	2.5–7.5
Creatinine in 24-h urine collection [mg/kg/24 h]	12.1	11–20
Alkaline phosphatase [U/L]	64	38–126
Vitamin D 25-OH [ng/mL]	39.2	>30
Parathyroid hormone PTH [pg/mL]	48.8	15–65
B-Cross Laps [pg/mL]	408.8	<1008
Osteocalcin [ng/mL]	21.7	15–46

**Table 2:** Results of bone densitometry (DXA).

BMD (g/cm^2^)	Date of Examination			
	4 April 2021	16 September 2021	13 December 2021	29 March 2022
Spine	0.782	0.769	0.762	0.789
Neck	0.728	–	0.677	0.697
Total Body	0.981	–	0.968	0.989

## Data Availability

Not applicable. All data are presented in the present manuscript.
